# Effects of Dietary Ratio of Insoluble Fiber to Soluble Fiber on Reproductive Performance, Biochemical Parameters, and Fecal Microbial Composition of Gestating Sows

**DOI:** 10.3390/ani15131850

**Published:** 2025-06-23

**Authors:** Xiaolu Wen, Qiwen Wu, Kaiguo Gao, Xuefen Yang, Hao Xiao, Zongyong Jiang, Li Wang

**Affiliations:** 1 Institute of Animal Science, Guangdong Academy of Agricultural Sciences, 1 Dafeng 1st Street, Guangzhou 510640, China; wenxiaolu@gdaas.cn (X.W.); wuqiwen@gdaas.cn (Q.W.); gaokaiguo@gdaas.cn (K.G.); yangxuefen@gdaas.cn (X.Y.); xiaohao@gdaas.cn (H.X.); jiangzy@gdaas.cn (Z.J.); 2 State Key Laboratory of Swine and Poultry Breeding Industry, 1 Dafeng 1st Street, Guangzhou 510640, China; 3 Key Laboratory of Animal Nutrition and Feed Science in South China, Ministry of Agriculture and Rural Affairs, 1 Dafeng 1st Street, Guangzhou 510640, China; 4 Guangdong Provincial Key Laboratory of Animal Breeding and Nutrition, 1 Dafeng 1st Street, Guangzhou 510640, China

**Keywords:** sow, soluble fiber, insoluble fiber, fecal microbial, reproductive performance

## Abstract

This study aimed to investigate the effects of dietary ISF:SF ratio on reproductive performance, biochemical parameters, colostrum composition, and fecal microbial composition in gestating sows. A total of 30 multiparous sows were randomly allocated to three dietary treatment groups: 8% inulin diet (ISF:SF 1.14, Inulin group), 8% cotton fiber diet (ISF:SF 6.61, Cotton group), and 4% inulin + 4% cotton fiber diet (ISF:SF 2.37, Inulin + Cotton group). The results showed that, compared to the other groups, the Inulin group had a significantly higher number of piglets born alive, as well as increased plasma concentrations of acetic acid, butyric acid, hexanoic acid, and total short-chain fatty acids (SCFAs) (*p* < 0.05). Sows in the Inulin group had significantly lower fecal scores than those in the other groups from days 81 to 85 and from days 106 to 110 of gestation (*p* < 0.05). On day 90 of gestation, the serum levels of albumin, urea, uric acid, calcium, and phosphorus in the Inulin group were significantly lower than those in the other groups (*p* < 0.05). Additionally, the serum levels of triacylglycerol in the Inulin + Cotton Fiber group were significantly higher than those in the other groups (*p* < 0.05). However, there were no significant differences in serum concentrations of total protein, creatinine, glucose, cholesterol, HDL-cholesterol, or LDL-cholesterol among the treatments (*p* > 0.05). On day 110 of gestation, the serum content of urea, uric acid, calcium, and phosphorus in the Inulin group was significantly lower than those in the other groups (*p* < 0.05). Furthermore, the plasma levels of uric acid, triacylglycerol, and HDL-cholesterol in the Inulin + Cotton Fiber group were significantly higher than those in the Cotton Fiber group (*p* < 0.05), while the creatinine levels in the Inulin group were higher than those in the other groups (*p* < 0.05). No differences were observed in the composition and immune performance of colostrum (*p* > 0.05). Microbial sequencing analysis showed that dietary inulin supplementation to increase the proportion of soluble fiber significantly decreased the abundance of Firmicutes, Clostridia, Clostridiales, Lachnospiraceae, Streptococcaceae, and *Streptococcus* (*p* < 0.05). The abundance of short-chain fatty acid-producing microorganisms—Bacteroidetes, Bacteroidia, Bacteroidales, and Muribaculaceae—was significantly increased (*p* < 0.05). The results indicated that inulin supplementation decreased the dietary ISF:SF ratio, significantly alleviated constipation in sows, increased the number of piglets born alive, regulated intestinal microecology, and increased the plasma concentrations of short-chain fatty acids (SCFAs), including acetic, propionic, and butyric acids.

## Simple Summary

This study aimed to investigate the effects of the dietary ratio of insoluble fiber to soluble fiber (ISF:SF) on the reproductive performance, biochemical parameters, and fecal microbial composition of gestating sows. The results indicated that inulin supplementation increased the ratio of dietary soluble to insoluble fiber, significantly alleviated constipation in sows, increased the number of piglets born alive, regulated intestinal microecology, and elevated the concentrations of short-chain fatty acids, including acetic acid, propionic acid, and butyric acid in plasma.

## 1. Introduction

Previous studies have confirmed that gestating sows can increase litter size by consuming a high-fiber diet [1]. Dietary fiber is characterized by its water-holding capacity, viscosity, and fermentability. Upon hydration, it swells, enhancing intestinal peristalsis, alleviating constipation in sows, increasing satiety, and reducing stereotypic behaviors, ultimately improving reproductive performance [2]. As a primary structural component of plant cell walls, dietary fiber consists of carbohydrates (e.g., pectin, cellulose, lignin) that resist digestion by mammalian endogenous enzymes but are metabolized by microbial communities in the porcine hindgut. Based on solubility, dietary fiber is classified into soluble fiber (e.g., pectin, arabinoxylans, inulin) and insoluble fiber (e.g., lignin, cellulose) [3]. Soluble fibers exhibit high digestibility and are rapidly fermented by hindgut microbiota to produce short-chain fatty acids (SCFAs) such as acetate and propionate [4,5]. In contrast, insoluble fibers enhance intestinal motility by stimulating mechanical activity, thereby accelerating digesta transit and reducing retention time within the intestinal lumen. Renteria-Flores et al. reported that sows fed diets rich in soluble fiber during pregnancy exhibited higher embryo survival rates and produced larger total litter sizes and live litter sizes compared to sows fed diets rich in insoluble fiber [6]. Other studies have also indicated that the addition of konjac powder and inulin to the diets of pregnant sows increases the proportion of soluble fiber, thereby enhancing their reproductive performance [7,8]. Li et al. further discovered that increasing the fiber content in the diets of pregnant sows significantly increases the feed intake during lactation, improves intestinal microecology, and raises the proportion of Lactobacillus [9]. Feyera et al. reported that dietary fiber supplementation in pregnant sows reduced the incidence of stillborn piglets and overall piglet mortality, as well as mortality due to poor viability and piglet diarrhea [10]. However, it has also been noted that increasing dietary fiber may not significantly affect the reproductive performance of sows [11]. The discrepancies in study results may stem from variations in the proportions of insoluble and soluble fiber in the diets. Nonetheless, the effects of these fiber proportions on the reproductive performance of pregnant sows remain inadequately explored. It is assumed that the ratio of insoluble fiber to soluble fiber in the diet affects the reproductive performance of sows. In this study, the ratio of insoluble to soluble fiber in the diets of sows was adjusted by incorporating inulin and cotton fiber. The effects of this ratio on reproductive performance, blood biochemical indices, and fecal microecology of sows were investigated.

## 2. Materials and Methods

The present experiment was conducted at the Research Farm of the Institute of Animal Science, Guangdong Academy of Agricultural Sciences, Guangzhou, China. The experimental protocol was approved by the Animal Care Committee of the Institute of Animal Science, Guangdong Academy of Agricultural Sciences, Guangzhou, P. R. China, with the approval number GAASISA-2019-035.

### 2.1. Swine and Diet

After breeding, a total of 30 multiparous (parity 3) Landrace × Yorkshire sows were assigned to three different dietary treatments based on body weight (BW) and backfat thickness. The three isoenergetic (2250 kcal NE/kg) and isonitrogenous (CP: 13.6%) gestation diets were formulated based on corn and soybean meal. The diets were as follows: (1) the first diet included 8% inulin (ISF:SF 1.14, Inu diet); (2) the second diet included 8% cotton fiber (ISF:SF 6.61, Cot diet); and (3) the third diet included 4% inulin and 4% cotton fiber (ISF:SF 2.37, Inu + Cot diet). These diets were designed to meet or exceed the nutrient requirements recommended by the National Research Council (NRC, 2012) [12]. The components of the three diets are presented in Table 1, and they contained similar levels of crude protein (CP) and digestible energy (DE). The animals were housed individually in gestation crates (2.1 × 0.7 m) before being moved to the farrowing room. They were fed twice daily at 7:30 and 16:30, receiving a total amount of 2.3 kg/d from days 0 to 30 of gestation, 2.5 kg/d from days 31 to 90 of gestation, and 2.8 kg/d from day 90 of gestation until farrowing. On day 110 of gestation, the sows were transported to farrowing stalls. After farrowing, all sows received the same standard lactation diet (3.41 Mcal/kg ME, 172.0 g/kg CP, 10.0 g/kg lysine).

### 2.2. Recording and Sampling

The backfat thickness of all sows was measured on days 30, 90, and 110 of gestation using an ultrasonic device (Renoc Lean-Meater, Digital Backfat Indicator, Minneapolis, MN, USA) at the P2 position (6 cm from the midline at the head of the last rib). After farrowing, the numbers of total born, born alive, stillborn, and mummified piglets were recorded. Individual piglet weights were measured at the time of farrowing. On days 90 and 110 of gestation, heparinized blood samples (5 mL) and non-anticoagulant blood were collected from the ear vein 12 h after feeding and centrifuged at 3000× *g* for 10 min at 4 °C to harvest plasma and serum samples, which were then stored at −80 °C until further analysis of plasma antioxidant index, short-chain fatty acids (SCFAs), biochemical indices, and immunoglobulin levels. On day 110 of gestation, fresh fecal samples were collected from the rectum of each sow using sterile cotton swabs. The samples were placed in sterile tubes and then immediately stored at −80 ° C for microbiota analysis. Within 2 h of the first piglet’s delivery, colostrum was collected from all functional teats after properly cleansing the udder with water. Approximately 30 mL of colostrum was collected into Falcon tubes and stored at −20 °C until analysis for milk composition and immunoglobulin content. During gestation days 81–85 and 106–110, a daily qualitative evaluation of the feces was conducted. Every morning before the daily cleaning, we ranked the feces of each sow by visual qualitative evaluation. Feces were scored as follows: 5 = absence of feces, 4 = dry and pellet-shaped, 3 = between dry and normal, 2 = normal and soft, but firm and well formed, 1 = between normal and wet, still formed but not firm, and 0 = very wet feces, unformed and liquid.

### 2.3. Chemical Analyses

The dietary crude protein was determined according to AOAC (method 2001.11), and crude fiber was analyzed according to AOAC (method 978.10), soluble and insoluble fiber levels were determined by AOAC (method 991.43) [13]. The concentrations of milk protein, milk fat, total solids, non-fat solids, and lactose in colostrum were measured using the Julie Z7 automatic analyzer (Scope Electric, Regensburg, Germany). Biochemical parameters, including total protein, creatinine, albumin, urea, uric acid, glucose, triacylglycerol, cholesterol, high-density lipoprotein (HDL) cholesterol, low-density lipoprotein (LDL) cholesterol, calcium, and phosphorus in serum, were analyzed with the Selectra Pro XL automatic biochemical analyzer (Vital Scientific, Spankeren, Gelderland, The Netherlands). An enzyme-linked immunosorbent assay (Nanjing Jiancheng Bioengineering Institute Co., Ltd., Nanjing, China) was performed to detect immunoglobulin A (IgA), immunoglobulin G (IgG), and immunoglobulin M (IgM) in colostrum and milk, following the manufacturer’s recommended protocol. Total antioxidant capacity (T-AOC), glutathione peroxidase (GSH-px), total superoxide dismutase (T-SOD), catalase (CAT), and malondialdehyde (MDA) were evaluated using commercial kits (Nanjing Jiancheng Bioengineering Institute Co., Ltd. (Nanjing, China). An Agilent 7890B gas chromatograph coupled with a 7000D mass spectrometer and equipped with a DB-FFAP column (30 m length × 0.25 mm i.d. × 0.25 μm film thickness, J&W Scientific, Santa Clara, CA, USA) was employed for GC-MS/MS analysis of short-chain fatty acids (SCFAs). Briefly, plasma samples were thawed and vortexed for 1 min prior to analysis. A total of 50 μL of samples were added to a 1.5 mL EP tube along with 100 μL of a 0.5% (*v*/*v*) phosphoric acid solution. The mixture was vortexed for 3 min, after which 150 μL of MTBE (containing the internal standard) was added. The internal standard working solution, containing 20 μg/mL acetic acid-d3, 20 μg/mL propionic acid-d5, 20 μg/mL butyric acid-d7, 8 μg/mL 2-methylvaleric acid, and 4 μg/mL isooctanoic acid-d15, was prepared using pure MTBE. The mixture was vortexed for an additional 3 min and then subjected to ultrasonication for 5 min. Subsequently, the mixture was centrifuged at 14,000× *g* for 10 min at 4 °C. The supernatant was collected and used for GC-MS/MS analysis. Helium was used as the carrier gas, at a flow rate of 1.2 mL/min. Injection was made in the split mode with a split ratio of 5:1, and the injection volume was 1 μL. The oven temperature was held at 50 °C for 1 min, raised to 220 °C at a rate of 18 °C/min, and held for 5 min. All samples were analyzed in multiple reaction monitoring mode. The injector inlet and transfer line temperatures were 250 °C and 230 °C, respectively.

### 2.4. Microbial Analyses

The total genomic DNA of fecal samples was extracted using the OMEGA Soil DNA Kit (Omega Bio-Tek, Norcross, GA, USA) according to the manufacturer’s instructions and stored at −20 °C. A Nanodrop ND-1000 spectrophotometer (Thermo Fisher Scientific, Waltham, MA, USA) was used to quantify the extracted DNA, and its quality was assessed using 1.2% agarose gel electrophoresis. The PCR amplification of the bacterial 16S rRNA gene V3-V4 regions was conducted with the forward primer 338F (5′-ACTCCTACGGGAGGCAGCAG-3′) and the reverse primer 806R (5′-TCGGACTACHVGGGTWTCTAAT-3′). The PCR reaction mixture included 4 μL of FastPfu Buffer (5×), 0.25 μL of Q5 DNA polymerase (5 U/μL), 2 μL of dNTPs (2.5 mmol/L), 0.8 μL of forward and reverse primers (5 µmol/L each), 0.2 μL of bovine serum albumin, 1 μL of DNA template (10 ng/μL), and 10.95 μL of ddH2O, resulting in a final volume of 20 μL. The thermal cycling conditions consisted of an initial denaturation step at 95 °C for 3 min, followed by 30 cycles of denaturation at 98 °C for 15 s, annealing at 55 °C for 30 s, and elongation at 72 °C for 45 s, concluding with a final extension at 72 °C for 10 min. The PCR product was extracted from a 2% agarose gel and purified using the AxyPrep DNA Gel Extraction Kit (Axygen Biosciences, Union City, CA, USA), followed by quantification with a Quantus™ Fluorometer (Promega, Madison, WI, USA). Sequencing was performed on the Illumina MiSeq PE300 platform (Shanghai Biozeron Biotech Co., Ltd., Shanghai, China).

### 2.5. Statistical Analysis

The differences in reproductive performance, SCFAs and blood biochemical index in plasma, antioxidant capacity of plasma, immune performance, and colostrum composition among the three treatment groups were analyzed using one-way ANOVA, followed by Tukey’s multiple comparison test, utilizing SPSS version 20.0 (SPSS Inc., Chicago, IL, USA). *p* < 0.05 was considered statistically significant, while a *p*-value of 0.05 ≤ *p* < 0.10 was regarded as a significant tendency. The α-diversity value and β-diversity value of fecal microorganisms were analyzed using QIIME2.0. The α-diversity index of the samples was determined based on the Shannon, Simpson, and Sobs indices. The β-diversity index was calculated using PCA, PCoA, and NMDS indices. The differences in α-diversity index, β-diversity index, and Specaccum were analyzed using R software (4.0.3). The Kruskal–Wallis test was employed to analyze microbial differences between groups at phylum, class, order, family, and genus levels. Correlations between differentiated bacteria and SCFAs were assessed using Spearman’s correlation test and GraphPad Prism version 5.00 (GraphPad Software, San Diego, CA, USA). Statistically significant differences are indicated with asterisks as follows: * *p* < 0.05,** *p* < 0.01,*** *p* < 0.001.

## 3. Result

### 3.1. Reproductive Performance of Sows

The effects of the ratio of insoluble fiber to soluble fiber in gestation diets on sow performance are presented in Table 2. The number of piglets born alive in the Inulin group was significantly higher than that in the Cotton fiber and Inulin + Cotton fiber groups (*p* < 0.05). There was a tendency for a higher number of total births in the Inulin group compared to the other groups (*p* = 0.08). No significant differences were observed in the number of stillborn or mummified piglets, litter weight of piglets born alive, average live birth weight, placenta weight, farrowing duration, birth intervals, or back fat thickness among the groups (*p* > 0.05).

### 3.2. Fecal Score of Sows

**Figure 1:**
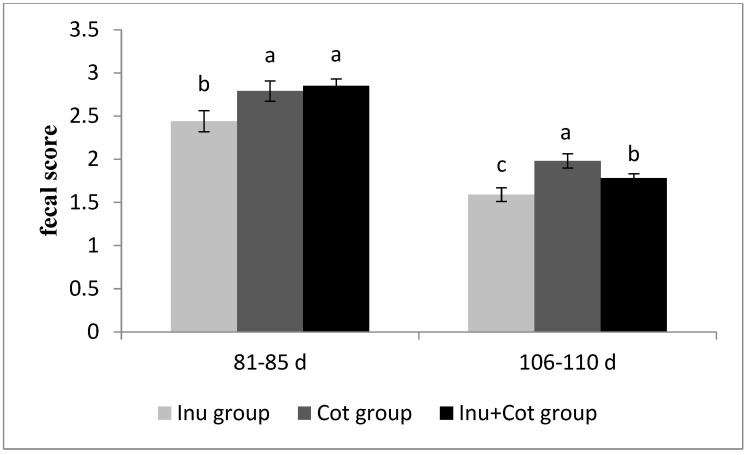
Effects of the ratio of insoluble fiber to soluble fiber in gestation diets on fecal score of sows. Feces were scored as follows: 5 = absence of feces, 4 = dry and pellet-shaped, 3 = between dry and normal, 2 = normal and soft, but firm and well formed, 1 = between normal and wet, still formed but not firm, and 0 = very wet feces, unformed and liquid. ^a–c^ Different letters denote significant differences (*p* < 0.05).

The effects of the ratio of insoluble fiber to soluble fiber in gestation diets on the fecal score of sows are shown in Figure 1. Between days 81 and 85 of gestation, the fecal score of the Inulin group sows was significantly lower than that of the other groups (*p* < 0.05). Between days 116 and 110 of gestation, the fecal score of the Inulin group sows was significantly lower than that of the other groups, and the Inulin + Cotton Fiber group was significantly lower than the Cotton Fiber group (*p* < 0.05).

### 3.3. Plasma SCFAs

The effects of the ratio of insoluble fiber to soluble fiber in gestation diets on plasma SCFAs of sows are shown in Table 3. The plasma concentrations of acetic acid, butyric acid, hexanoic acid, and total SCFAs in the Inulin group were significantly higher than those of the other groups (*p* < 0.05). The plasma concentrations of acetic acid and total SCFAs in the Inulin + Cotton Fiber group were significantly higher than those of the Cotton Fiber group (*p* < 0.05). There were no significant differences in propionic acid, isobutyric acid, isovaleric acid, or valeric acid between all groups.

### 3.4. Serum Biochemical Index

The effects of the ratio of insoluble fiber to soluble fiber in gestation diets on the serum biochemical index of sows are shown in Table 4. At day 90 of gestation, the content of albumin, urea, uric acid, calcium, and phosphorus in the serum of the Inulin group was significantly lower than those in other groups (*p* < 0.05), and the content of triacylglycerol in the serum of the Inulin + Cotton Fiber group was significantly higher than those in other groups (*p* < 0.05). However, there were no differences in serum total protein, creatinine, glucose, cholesterol, HDL-cholesterol, or LDL-cholesterol concentrations among treatments (*p* > 0.05). At day 110 of gestation, the content of uric acid, calcium, and phosphorus in the serum of the Inulin group were significantly lower than those in other groups (*p* < 0.05), the content of uric acid, triacylglycerol, and HDL-cholesterol in the serum of the Inulin + Cotton Fiber group was significantly higher than that in the Cotton Fiber group (*p* < 0.05), and the content of creatinine in Inulin group was higher than that in other groups (*p* < 0.05). However, there were no differences in serum total protein, albumin, urea, glucose, cholesterol, or LDL-cholesterol concentrations among treatments (*p* > 0.05).

### 3.5. Colostrum Composition

The effects of the ratio of insoluble fiber to soluble fiber in gestation diets on the composition of colostrum are shown in Table 5. The ratio of insoluble fiber to soluble fiber in gestation diets had no significant effect on the concentration of milk protein, milk fat, total solid, non-fat solid, lactose, IgA, IgM, or IgG of colostrum in sows (*p* > 0.05).

### 3.6. Plasma Antioxidant Capacity

The effects of the ratio of insoluble fiber to soluble fiber in gestation diets on the plasma antioxidant capacity of sows are shown in Table 6. At day 90 gestation, the activity of plasma CAT in the Inulin group was significantly higher than that of the other groups (*p* < 0.05), and the plasma MDA content of the Cotton Fiber group was higher than that of the other groups. There were no significant differences in the activity of T-SOD and T-AOC in plasma among treatments (*p* > 0.05).

At day 110 gestation, the plasma T-AOC content of the Inulin group was significantly higher than that of the Inulin + Cotton Fiber group (*p* < 0.05). There were no differences in T-SOD activity, CAT activity, or MDA content in plasma among treatments (*p* > 0.05).

### 3.7. Fecal Microbiota

**Figure 2:**
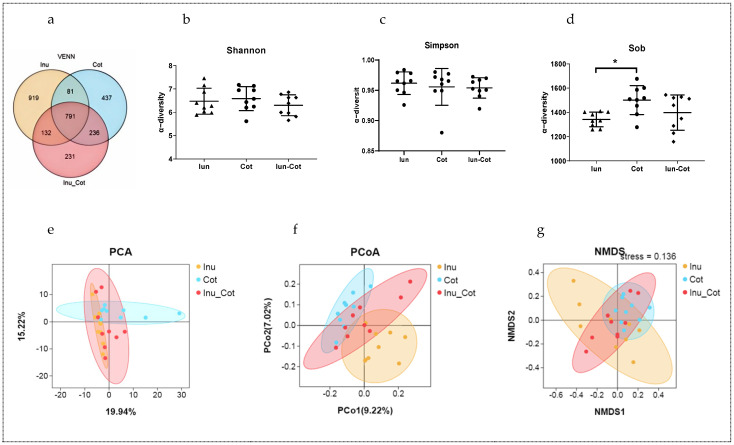
Effects of the ratio of insoluble fiber to soluble fiber in gestation diets on the fecal microbial diversity of sows. Venn diagram showed the proportion of common and special OTUs among groups (**a**). α−diversity indices such as Shannon (**b**), Simpson (**c**), and Sobs (**d**) indices indicated the diversity and evenness. β−diversity indices such as PCA (**e**), PcoA (**f**), and NMDS (**g**) indices were used to display the distribution of the samples among groups. Statistically significant differences are indicated with asterisks as follows: * *p* < 0.05.

The Venn diagram in Figure 2a shows the proportion of common and special operational taxonomic units (OUTs) among groups. There were 919, 437, and 231 special OUTs in the Iunlin, Cotton Fiber, and Inulin + Cotton Fiber groups, respectively, and a total of 791 shared OTUs in the three groups. As shown in Figure 2d, the Sobs index was higher in fecal samples of the Cotton Fiber group compared with the Inulin group (*p* < 0.05). Shannon and Simpson indices in Figure 2b,c show that there is no significant difference between all groups (*p* > 0.05). PCA (Figure 2e), PCoA (Figure 2f), and NMDS (Figure 2g) plots to weighted UniFrac distance were applied to evaluate differences in β-diversity. The PCoA plot indicated that the ratio of insoluble fiber to soluble fiber in gestation diets had an effect on the gut microbial structure of sows.

**Figure 3:**
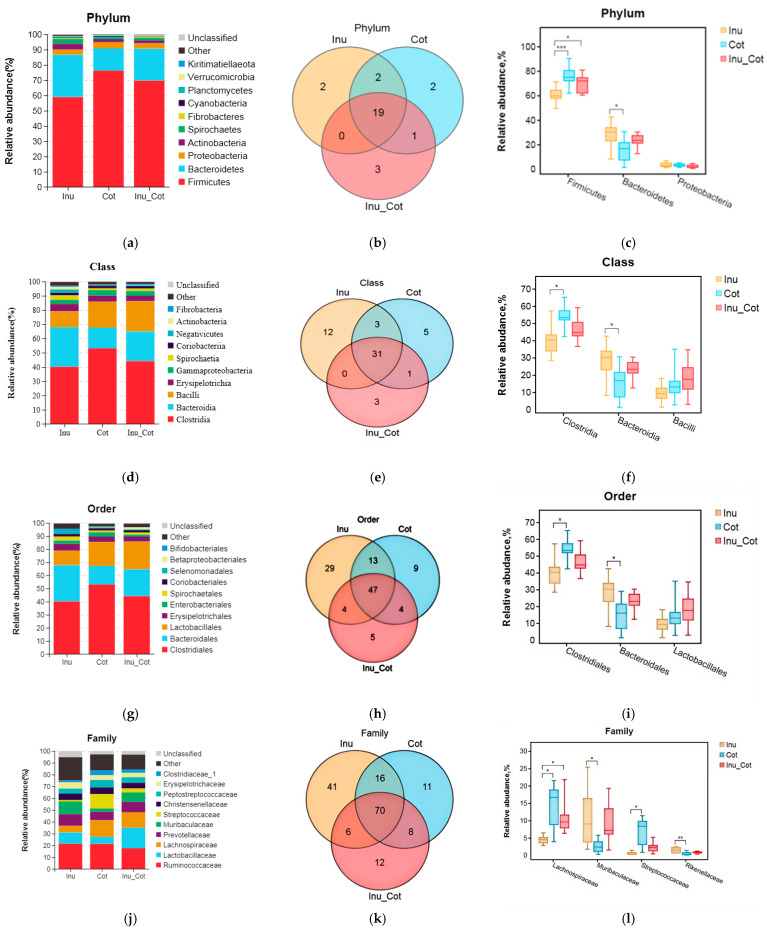

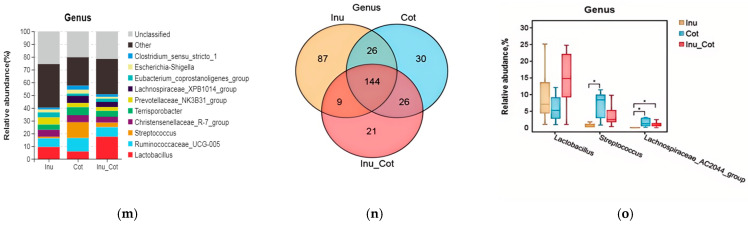
Effects of the ratio of insoluble fiber to soluble fiber in gestation diets on fecal microbiota composition of sows. The relative abundance of the top ten phyla (**a**), classes (**d**), orders (**g**), families (**j**), and genera (**m**) are shown. The Venn diagram showed the proportion of common and special phyla (**b**), classes (**e**), orders (**h**), families (**k**), and genera (**n**) among groups. The significantly different microbial at each level are shown: phylum (**c**), class (**f**), order (**i**), family (**l**), and genus (**o**). Statistically significant differences are indicated with asterisks as follows: * *p* < 0.05, ** *p* < 0.01,*** *p* < 0.001.

The composition of the top 10 phyla (Figure 3a), classes (Figure 3d), orders (Figure 3g), families (Figure 3j), and genera (Figure 3m) were provided. Results indicated that Firmicutes, Bacteroidetes, Proteobacteria, Actinobacteria, and Spirochaetes were major phyla of fecal microbiota of sows. Firmicutes relative abundance of the Inulin group was significantly lower than that of other groups, and the Bacteroidetes relative abundance was higher than the Cotton Fiber group (*p* < 0.05). At the class level, the most prevalent microbes were Clostridia, Bacteroidia, Bacilli, Erysipelotrichia, and Gammaproteobacteria. The relative abundance of Clostridia in the Inulin group was lower than in the Cotton Fiber group (*p* < 0.05), and Bacteroidia was higher than in the Cotton Fiber group (*p* < 0.05). At the order level, Clostridiales, Bacteroidales, Lactobacillales, Erysipelotrichales, and Enterobascteriales were the main orders in fecal microbiota. The relative abundance of Clostridiales in the Inulin group was lower than in the Cotton Fiber group (*p* < 0.05), and Bacteroidales was higher than in the Cotton Fiber group (*p* < 0.05). At the family level, the major fecal microbiota of sows were Ruminococcaceae, Lactobacillaceae, Lachnospiraceae, Prevotellaceae, and Muribaculaceae. The relative abundances of Lachnospiraceae and Streptococcaceae in the Inulin group were lower than in the Cotton Fiber group (*p* < 0.05), and those of Muribaculaceae and Rikenellaceae were higher than Cotton Fiber group (*p* < 0.05). At the genus level, the major fecal microbiota of sows were *Lactobacillus*, *Ruminococcaceae_UCG-005*, *Streptococcus*, *Christensenellaceae_R-7_group*, and *Temisporobacter.* The relative abundance of *Streptococcus* and *Lachnospiraceae_AC2044_group* in the Inulin group was lower than those in the Cotton Fiber group (*p* < 0.05).

### 3.8. Associations of Differential Fecal Microbes with the Plasma SCFA

**Figure 4:**
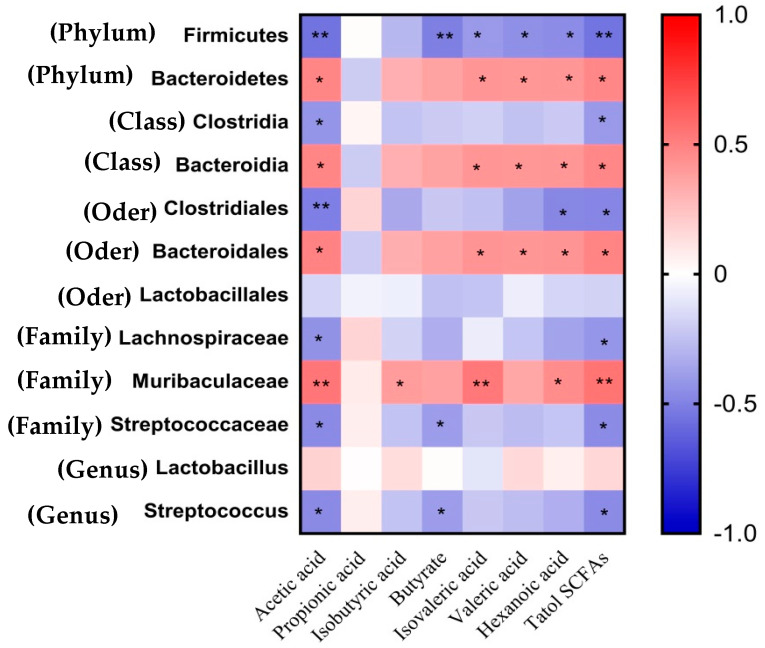
The Spearman correlation analysis between significantly differential microbes and plasma concentration of SCFAs. In the heatmap of the correlation coefficient, the red represents positive correlation, and the blue represents negative correlation, respectively (* *p* < 0.05, ** *p* < 0.01).

As displayed in Figure 4, negative associations between Firmicutes (phylum) and the plasma concentrations of acetic acid, butyric acid, isovaleric acid, valeric acid, hexanoic acid, and total SCFAs were observed (*p* < 0.05). Bacteroidetes (phylum), Bacteroidia (*class*), and Bacteroidales (*order*) were positively (*p* < 0.05) correlated with the plasma concentrations of acetic acid, isovaleric acid, valeric acid, hexanoic acid, and total SCFAs. The Muribaculaceae (family) was positively (*p* < 0.05) correlated with acetic acid, isobutyric acid, isovaleric acid, hexanoic acid, and total SCFAs. Clostridia (*class*) and Lachnospiraceae (family) were negatively (*p* < 0.05) correlated with the plasma concentrations of acetic acid and total SCFAs.

In addition, the plasma concentrations of acetic acid, butyric acid, and total SCFAs were negatively (*p* < 0.05) associated with Streptococcaceae (family) and *Streptococcus* (genus) (*p* < 0.05), while concentrations of acetic acid, hexanoic acid, and total SCFAs showed the same associations with Clostridiales (order) (*p* < 0.05).

## 4. Discussion

Recent studies have reported that increasing fiber intake during gestation improves both the litter size of sows and their feed intake. In this study, we explored the ratio of insoluble fiber to soluble fiber in gestation diets and its impact on sow reproductive performance and fecal microbial composition. A significantly higher number of piglets born alive was observed when the ratio of insoluble fiber to soluble fiber was 1.14. There was also a tendency for a higher number of total births in the Inulin group compared to the other groups. These findings suggest that increasing the proportion of soluble fiber in the feed enhances the reproductive performance of sows. Other studies have shown that increasing the proportion of soluble fiber in the diet of pregnant sows increases the number of piglets born alive and feed intake during lactation [1,14]. Sun et al. reported that increasing the proportion of dietary soluble fiber by adding konjac powder to the diet increased the number of live piglets produced by sows and significantly improved the weaning weight of piglets [7]. Li et al. found that inulin supplementation reduced the ratio of insoluble to soluble fiber in the diets of pregnant sows, increased the weaning weight of piglets, and improved the intestinal mucosa morphology of piglets, although it had no significant effect on the litter size of sows [15]. Tan et al. reported that when konjac powder was fed over two consecutive breeding cycles, the results showed that increasing the proportion of soluble fiber in the diet significantly increased the number of weaned piglets, weaned litter weight, daily gain of piglets, and feed intake of sows during lactation in the second breeding cycle, but had no significant effect on reproductive performance in the first breeding cycle [16]. Increasing the dietary soluble fiber level significantly reduced the number of intrauterine growth-restricted (IUGR) piglets and significantly improved the uniformity survival rate of embryos in piglets [6,17]. Vestergaard reported that dietary supplementation with a high content of soluble fiber negatively affected piglet birth weight [18]. Other studies have also indicated that reducing the dietary insoluble to soluble fiber ratio from 8.1 to 3.2 had no significant effect on the number of piglets born alive and the birth weight of piglets, but significantly reduced the weight gain of piglets during lactation [11]. The reasons for these discrepancies may stem from variations in the dietary ratios of insoluble to soluble fiber and the differing durations of diet administration. The ratio of insoluble to soluble fiber in the diet affects nutrient digestibility [19]. In this study, the ratio of insoluble to soluble fiber in the diets ranged from 1.14 to 6.61, which was lower than that reported in other studies. In this study, sows were fed the experimental diet on the 30th day of gestation, whereas Liu et al. initiated their experiment on the 90th day of gestation. The increase in live litter size may be attributed to the fact that soluble fiber alleviates constipation and reduces the risk of dystocia in sows [20].

Constipation is a common concern in sow breeding, particularly during the post-pregnancy and pre-lactation phases. The primary contributing factors include the following: (1) limited space in pens, which restricts the movement of sows; (2) feeding restrictions that result in inadequate feed intake and prolonged intestinal retention of digesta; (3) the rapid growth of the fetus during late pregnancy, which enlarges the uterus, increases intestinal pressure, and reduces intestinal peristalsis; (4) insufficient water intake, particularly in hot weather; and (5) increased intestinal water reabsorption due to the demands of lactation [21,22]. Constipation can prolong labor, potentially leading to dystocia and an increased incidence of stillbirth. Additionally, it may increase the absorption of endotoxins in the intestine, contributing to mastitis and postpartum agalactia syndrome. Reduced feed intake and consequently diminished lactation performance can negatively affect the reproductive performance of sows [23]. In this study, increasing soluble fiber content in the diet significantly reduced fecal scores in sows, alleviating constipation. Our results were consistent with other studies, which showed that increasing the proportion of soluble fiber reduced constipation in sows by regulating intestinal microecology [24]. Soluble fiber is highly hydrated and fermentable, which increases fecal water content and softens feces; short-chain fatty acids produced by fermentation stimulate intestinal smooth muscle contraction. Gases such as carbon dioxide, hydrogen, and methane produced by the fermentation of soluble dietary fiber can also stimulate intestinal peristalsis and promote defecation [2,25]. In this study, we also found that the fecal scores at 106–110 days of gestation were generally lower than those at 81–85 days. This might be due to the increased feed intake in the later stage of gestation, which promotes intestinal peristalsis.

SCFAs are the“bridge” between diet, gut microbiota, and the host. They play a significant role in regulating cholesterol and fat synthesis in the liver, as well as stabilizing blood glucose levels by stimulating glucagon secretion and enhancing satiety. SCFAs inhibit fat deposition and modulate intestinal inflammation in sows, demonstrating a potential positive regulatory effect in pregnant sows [26,27]. In this study, we found that the plasma concentrations of acetic acid, butyric acid, hexanoic acid, and total SCFAs in the Inulin group were significantly higher than those in the other groups. As the dietary ratio of insoluble fiber to soluble fiber decreased, the plasma SCFA concentrations increased significantly. This finding aligns with a previous study [16], which reported that reducing the ratio of insoluble fiber to soluble fiber by adding konjac flour and beet pulp significantly increased SCFA concentrations. Additionally, other studies [28,29] reported a significant increase in fecal concentrations of acetic acid, propionic acid, butyric acid, and total SCFAs when the ratio of insoluble fiber to soluble fiber decreased from 6.00 to 4.00 or from 3.89 to 1.81.

Serum biochemical indices indicate alterations in tissue cell permeability and metabolism, reflecting the overall health and nutritional status of animals [30]. In the present study, the serum urea and uric acid concentrations of sows in the inulin group were significantly lower than those in the other groups. This finding suggests that reducing the ratio of insoluble to soluble fiber in the diet could enhance the efficiency of amino acid utilization, which aligns with previous studies [31]. Soluble fibers are generally more fermentable than insoluble fibers. Consequently, fiber fermentation increases the bacterial population, leading to a significant conversion of ammonia into bacterial proteins [32]. This process subsequently reduces the uptake of urea nitrogen into the bloodstream. The serum concentrations of calcium (Ca) and phosphorus (P) indicated that the absorption of these minerals from the diet was inhibited by a decreasing dietary ratio of insoluble to soluble fiber. This reduction in mineral utilization may be attributed to the strong cation exchange capacity of soluble fiber in the diet, which can adsorb cations such as calcium and phosphorus [33]. An intriguing finding in this study was that as the dietary ratio of insoluble fiber to soluble fiber increased, the concentrations of HDL-cholesterol and triglycerides in serum initially rose before subsequently declining. Triglycerides and HDL cholesterol are important indicators of lipid metabolism. Our results suggest that the dietary ratio of insoluble to soluble fiber affects lipid metabolism, which is consistent with previous studies [34,35].

In late gestation, the rapid development of the fetus increases the metabolic demands of sows, leading to elevated production of reactive oxygen species (ROS). Accumulated ROS that are not efficiently cleared can damage intracellular lipids and proteins, thereby inducing oxidative stress [36]. Oxidative stress negatively impacts the reproductive performance of sows, primarily manifested as reduced litter size and impaired lactation capacity. In this study, dietary soluble fiber was found to increase plasma catalase (CAT) concentration and decrease malondialdehyde (MDA) concentration in sows on day 90 of gestation, suggesting enhanced antioxidant capacity. Notably, the plasma total antioxidant capacity (T-AOC) was significantly higher in the Inulin group compared to the Inulin + Cotton group. CAT and superoxide dismutase (SOD) are critical enzymes that play vital roles in combating oxidative stress. T-AOC serves as a comprehensive indicator of the body’s overall antioxidant capacity [37]. Conversely, MDA, a byproduct of lipid peroxidation, is closely associated with cellular damage and is widely used as a biomarker for the severity of oxidative stress [38]. Supporting these findings, Li et al. demonstrated that inulin supplementation increased the soluble-to-insoluble fiber ratio, thereby enhancing plasma total SOD and T-AOC activities, which improved antioxidant performance in sows [14]. Similarly, Wang et al. (2016) reported that dietary inulin elevated plasma T-SOD and glutathione peroxidase (GSH-Px) activities while reducing MDA levels [39]. The underlying mechanism may involve soluble fiber upregulating nuclear factor erythroid 2-related factor 2 (Nrf2) and heme oxygenase-1 (HO-1), which are key regulators of endogenous antioxidant enzymes [14,40].

The gut microbiota plays a crucial role in host health by promoting the development of the immune system, reducing inflammation, and competitively suppressing pathogens [41]. Pregnant sows, being in a unique physiological state, often encounter issues such as constipation, dystocia, and excessive weight gain. Recent studies suggest a strong association between constipation and gut microbes [42]. This study revealed a significant difference in the microbial community structure of sows in the Inulin group compared to the Cotton group on day 110 of gestation. Furthermore, the diet of the Cotton group was found to enhance the α-diversity index of fecal microbiota in sows. Research indicates that the proportions of Bacteroidetes and Firmicutes in the gut decrease during episodes of constipation [43], which aligns with the findings of this study. An increase in dietary insoluble fiber was found to reduce Bacteroidetes levels while increasing Firmicutes levels, as well as the constipation coefficient in sows. Moreover, the Inulin group also reduced the relative abundance of fecal Clostridia, Clostridiales, and Streptococcus, which are associated with intestinal inflammation [44]. Other studies have reported that increasing the ratio of soluble fiber to insoluble fiber in the diet can reduce the relative abundance of Streptococcus [8]. Correlation analysis showed that these bacteria were significantly negatively correlated with acetic acid, butyric acid, and total SCFAs. In addition, the Inulin group increased the relative abundance of Bacteroidia, Bacteroidales, Muribaculaceae, and Rikenellaceae in feces. Correlation analysis indicated that these bacteria were significantly positively correlated with acetic acid, isovaleric acid, valeric acid, hexanoic acid, and total SCFAs.

## 5. Conclusions

The results indicated that inulin supplementation increased the ratio of dietary soluble to insoluble fiber, significantly alleviated constipation in sows, increased the number of piglets born alive, regulated intestinal microecology, and elevated the concentrations of short-chain fatty acids, including acetic acid, propionic acid, and butyric acid in plasma.

## Figures and Tables

**Table 1:** Composition and nutrient level of the diets for gestating sows (air-dry basis).

Ingredient	8% Inulin	8% Cotton Fiber	4% Inulin + 4% Cotton Fiber
Corn	69.85	69.85	69.85
Soybean meal	18.00	18.00	18.00
Inulin	8.00	-	4.00
Cotton fiber powder	-	8.00	4.00
L-Lysine-HCl	0.10	0.10	0.10
Dicalcium phosphate	1.20	1.20	1.20
Limestone	1.20	1.20	1.20
Salt	0.40	0.40	0.40
Choline chloride (50%)	0.25	0.25	0.25
Premix ^a^	1.00	1.00	1.00
Total	100.00	100.00	100.00
Calculated nutritional level ^b^			
Digestible energy, kcal/kg	3077	3077	3077
Net energy, kcal/kg	2250	2250	2250
Crude protein, %	13.60	13.60	13.60
Crude fiber, %	9.75	9.75	9.75
Soluble fiber, %	8.68	2.28	5.48
Insoluble fiber, %	9.75	16.15	12.95
Lysine, %	0.79	0.79	0.79
Methionine + Cysteine, %	0.50	0.50	0.50
Threonine, %	0.53	0.53	0.53
Calcium, %	0.78	0.78	0.78
Phosphorus, %	0.50	0.50	0.50
STTD Phosphorus, %	0.32	0.32	0.32
Analyzed nutritional level			
Crude protein, %	13.71	13.52	13.65
Crude fiber, %	9.45	9.50	9.66
Soluble fiber, %	8.50	2.45	5.46
Insoluble fiber, %	9.65	16.20	12.95
Insoluble fiber:Soluble fiber	1.14	6.61	2.37

^a^ Provide the following per kg complete diet: vitamin A, 12,000 IU; vitamin D_3_, 2400 IU; vitamin E, 44 IU; vitamin K, 2 mg; biotin, 1 mg; folic acid, 5 mg; vitamin B_1_, 4 mg; vitamin B_2_, 15 mg; vitamin B_6_, 5 mg; vitamin B_12_, 50 μg; niacin, 30 mg; pantothenic acid, 40 mg; choline chloride, 800 mg; Fe (ferrous sulfate), 100 mg; Cu (copper sulfate), 12 mg; Mn (manganese sulfate), 25 mg; Zn (zinc sulfate), 120 mg; I (calcium iodate), 0.2 mg; Se (sodium selenite), 0.2 mg. ^b^ Calculated chemical concentrations using nutritional values for feed ingredients from the NRC (2012). STTD: standardized total tract digestibility.

**Table 2:** Effects of the ratio of insoluble fiber to soluble fiber in gestation diets on reproductive performance of sows.

Item	Inulin	Cotton Fiber	Inulin + Cotton Fiber	SEM	*p*-Value
Total born, *n*	13.33	12.30	12.44	0.460	0.080
Born alive, *n*	13.11 ^a^	11.50 ^b^	11.89 ^b^	0.477	0.045
Stillborn, *n*	0.33	0.30	0.44	0.143	0.940
Mummified, *n*	0.38	0.10	0.22	0.170	0.237
Litter weight of born alive, kg	18.84	17.18	17.55	0.708	0.769
Average live birth weight, kg	1.44	1.41	1.42	0.091	0.963
Weight of placenta, kg	4.46	3.98	3.72	0.223	0.407
Farrowing duration, min	223	189	224	15.590	0.589
Birth intervals, min	17.00	15.98	17.05	1.680	0.651
Back fat, cm					
Day 30	2.21	2.23	2.34	0.093	0.786
Day 90	2.20	2.25	2.30	0.074	0.456
Day 110	2.17	2.33	2.29	0.082	0.414

SEM: standard error of the mean. ^a,b^ Means within a row with different superscripts indicate significant differences (*p* < 0.05).

**Table 3:** Effects of the ratio of insoluble fiber to soluble fiber in gestation diets on plasma SCFAs of sows.

Item	Inulin	Cotton Fiber	Inulin + Cotton Fiber	SEM	*p*-Value
Acetic acid, μg/L	2934 ^a^	1229 ^c^	1893 ^b^	126	0.001
Propionic acid, μg/L	383	417	430	14	0.413
Isobutyric acid, μg/L	33.19	15.26	21.70	3.83	0.142
Butyric acid, μg/L	276.00 ^a^	73.03 ^b^	132.75 ^b^	23.25	0.001
Isovaleric acid, μg/L	41.16	23.28	27.79	4.14	0.180
Valeric acid, μg/L	14.28	9.74	11.89	1.05	0.197
Hexanoic acid, μg/L	64.54 ^a^	37.79 ^b^	43.69 ^b^	3.69	0.001
Total-SCFAs, μg/L	3746.17 ^a^	1805.1 ^c^	2560.82 ^b^	144	0.001

SEM: standard error of the mean. ^a–c^ Means within a row with different superscripts indicate significant differences (*p* < 0.05).

**Table 4:** Effects of the ratio of insoluble fiber to soluble fiber in gestation diets on the serum biochemical indices of sows.

Item	Inulin	Cotton Fiber	Inulin + Cotton Fiber	SEM	*p*-Value
90 day					
Total protein, g/L	74.78	67.41	74.12	2.411	0.315
Creatinine, μmol/L	235.02	206.78	226.32	5.24	0.072
Albumin, g/L	32.60 ^b^	37.93 ^a^	39.23 ^a^	1.005	0.013
Urea, mmol/L	12.03 ^b^	17.04 ^a^	17.43 ^a^	1.081	0.001
Uric acid, μmol/L	19.32 ^b^	22.35 ^a^	25.73 ^a^	1.084	0.001
Glucose, mmol/L	3.66	3.66	3.95	0.086	0.306
Triacylglycerol, mmol/L	1.47 ^b^	1.62 ^b^	1.95 ^a^	0.069	0.011
Cholesterol, mmol/L	1.44	1.54	1.55	0.045	0.593
HDL—Cholesterol, mmol/L	3.79	4.43	4.19	0.161	0.278
LDL—Cholesterol, mmol/L	0.68	0.70	0.69	0.026	0.953
Calcium, mmol/L	2.45 ^b^	2.54 ^a^	2.73 ^a^	0.028	0.001
Phosphorus, mmol/L	1.68 ^b^	1.85 ^a^	1.83 ^a^	0.023	0.001
110 day					
Total protein, g/L	70.89	73.26	71.86	1.469	0.435
Creatinine, μmol/L	236.25 ^a^	205.88 ^b^	213.35 ^b^	4.795	0.02
Albumin, g/L	41.60	42.06	36.82	1.074	0.097
Urea, mmol/L	17.53	18.07	18.43	0.431	0.722
Uric acid, μmol/L	30.90 ^c^	36.55 ^b^	44.39 ^a^	1.427	0.001
Glucose, mmol/L	4.03	4.00	4.16	0.094	0.776
Triacylglycerol, mmol/L	2.46 ^a,b^	2.01 ^b^	2.97 ^a^	0.155	0.036
Cholesterol, mmol/L	1.41	1.44	1.41	0.041	0.929
HDL—Cholesterol, mmol/L	4.84 ^a,b^	4.47 ^b^	5.34 ^a^	0.146	0.05
LDL—Cholesterol, mmol/L	0.62	0.64	0.61	0.025	0.857
Calcium, mmol/L	2.81 ^b^	2.97 ^a^	3.07 ^a^	0.033	0.003
Phosphorus, mmol/L	1.62 ^b^	1.92 ^a^	1.84 ^a^	0.041	0.003

SEM: standard error of the mean. ^a–c^ Means within a row with different superscripts indicate significant differences (*p* < 0.05).

**Table 5:** Effects of the ratio of insoluble fiber to soluble fiber in gestation diets on the composition of colostrum in sows.

Item	Inulin	Cotton Fiber	Inulin + Cotton Fiber	SEM	*p*-Value
Milk protein, %	15.04	15.81	17.13	0.571	0.344
Milk fat, %	5.72	5.34	6.46	0.236	0.140
Total solid, %	27.46	27.75	30.50	0.690	0.147
Non-fat solid, %	19.24	19.83	20.60	0.487	0.552
Lactose, %	4.01	3.76	3.51	0.117	0.229
IgA, μg/mL	14.92	12.66	14.02	0.98	0.653
IgM, μg/mL	25.30	24.21	25.04	0.42	0.539
IgG, μg/mL	192.36	189.00	180.76	8.38	0.850

SEM: standard error of the mean.

**Table 6:** Effects of the ratio of insoluble fiber to soluble fiber in gestation diets on the plasma antioxidant capacity of sows.

Item	Inulin	Cotton Fiber	Inulin + Cotton Fiber	SEM	*p*-Value
90 day					
T-AOC, mmol/L	0.32	0.31	0.32	0.007	0.643
T-SOD, U/mL	396	408	406	8.320	0.817
CAT, U/mL	2.68 ^a^	1.66 ^b^	1.90 ^b^	0.165	0.025
MDA, nmol/mL	2.57 ^b^	3.59 ^a^	1.70 ^c^	0.180	0.001
110 day					
T-AOC, mmol/L	0.26 ^a^	0.25 ^a,b^	0.23 ^b^	0.005	0.005
T-SOD, U/mL	434	394	346	14.960	0.059
CAT, U/mL	1.93	1.65	2.06	0.113	0.153
MDA, nmol/mL	1.26	1.32	1.20	0.065	0.769

SEM: standard error of the mean. ^a–c^ Means within a row with different superscripts indicate significant differences (*p* < 0.05).

## Data Availability

All the datasets used and analyzed during the current study are included in the manuscript.

## Abbreviations

NO: nitric oxide; SID, standardized ileal digestible; DE, digestible energy; UN, urea nitrogen; DM, dry matter; HDL, high-density lipoprotein; LDL, low-density lipoprotein; NLIN, non-linear regression; CP, crude protein; ISF, insoluble fiber; SF, soluble fiber; ROS, reactive oxygen species.
